# Understanding equity of institutional delivery in public health centre by level of care in India: an assessment using benefit incidence analysis

**DOI:** 10.1186/s12939-020-01331-z

**Published:** 2020-12-09

**Authors:** Sanjay K. Mohanty, Radhe Shyam Mishra, Suyash Mishra, Soumendu Sen

**Affiliations:** 1 Department of Fertility Studies, International Institute for Population Sciences, Mumbai, India; 2 Research Scholar, International Institute for Population Sciences, Mumbai, India

**Keywords:** Delivery care, Benefit incidence, Equity, National Health Mission, India

## Abstract

**Background:**

The National Health Mission (NHM), the largest ever publicly funded health programme worldwide, used over half of the national health budget in India and primarily aimed to improve maternal and child health in the country. Though large scale public health investment has improved the health care utilization and health outcomes across states and socio-economic groups in India, little is known on the equity concern of NHM. In this context, this paper examines the utilization pattern and net benefit of public subsidy for institutional delivery by the level of care in India.

**Methods:**

Data from the most recent round of the National Family Health Survey (NFHS 4), conducted during 2015–16, was used in the study. A total of 148,645 last birth delivered in a health centre during the 5 years preceding the survey were used for the analyses. Out-of-pocket (OOP) payment on delivery care was taken as the dependent variable and was analysed by primary care and secondary level of care. Benefits Incidence Analysis (BIA), descriptive statistics, concentration index (CI), and concentration curve (CC) were used to do the analysis.

**Results:**

Institutional delivery from the public health centres in India is pro-poor and has a strong economic gradient. However, about 28% mothers from richest wealth quintile did not pay for delivery in public health centres compared to 16% among the poorest wealth quintile. Benefit incidence analyses suggests a pro-poor distribution of institutional delivery both at primary and secondary level of care. In 2015–16, at the primary level, about 32.29% of subsidies were used by the poorest, 27.22% by poorer, 20.39% by middle, 13.36% by richer and 6.73% by the richest wealth quintile. The pattern at the secondary level was similar, though the magnitude was lower. The concentration index of institutional delivery in public health centres was − 0.161 [95% CI, − 0.158, − 0.165] compared to 0.296 [95% CI, 0.289, 0.303] from private health centres.

**Conclusion:**

Provision and use of public subsidy for institutional delivery in public health centres is pro-poor in India. Improving the quality of service in primary health centres is recommended to increase utilisation and reduce OOP payment for health care in India.

**Supplementary Information:**

The online version contains supplementary material available at 10.1186/s12939-020-01331-z.

## Introduction

Increasing health spending and rising health inequality are concomitant across geographies and socio-economic groups [1–4]. Rising health spending is associated with increased public investment in health and declining out-of-pocket (OOP) payments [5, 6]. Despite the increased public investment, catastrophic health spending (CHS) and impoverishment resulting from OOP payment have been increasing in many developing countries [7–9]. CHS, and impoverishment, due to health spending vary across countries and depend on income level, public policies, coverage of health insurance schemes, type of provider, payment methods, disease burden and demographics [10–12]. Globally about 1.3 billion people do not have access to effective and affordable health care. Of those who do have access, about 170 million are forced to spend more than 40% of their household income on medical treatment. Over 100 million people are pushed into extreme poverty due to health spending annually [13].

Equity and efficiency are two pillars of public health investment worldwide. Goal 4 and 5 of the Millennium Development Goals (MDGs) and goal 3 and 10 of the Sustainable Development Goals (SDGs) outlined the specific goals to reduce inequality in access and utilization to quality health services [14, 15]. Goal 3.7 aimed to achieve universal access to sexual and reproductive health-care services, while Goal 3.8 aim to achieve universal health coverage, financial risk protection, and access to quality health services by 2030. The progress in access to these services, measured by the universal health index (UHI) of service coverage based on reproductive health, nutrition, new-born and child health, infectious diseases, non-communicable diseases and service capacity and access among the general and most advantages population is slow and uneven across and within countries. Financial protection, as measured by catastrophic health spending, a key impediment in access to health services, has increased from 9.7% in 2000 to 11.7% by 2010 [7] and then, also increased in impoverishment due to the medical expenditure [16].

Many welfare governments have made large-scale investments to increase the access and utilisation of health care services. Periodic evaluation suggests a mixed impact of public health investment on health care utilization and health outcomes [17–20]. Public subsidy for health care increases utilization and reduced inequality in access to it [21, 22]. The equity impact of the public subsidy varies by the level of care (primary health centre and hospital) and the type of services (inpatient and outpatient) [17, 20, 23]. In most of the African countries, the distribution of public subsidy benefits the rich more the than poor (it is pro-rich) irrespective of the level of care [21, 24, 25], while in Asia, varying pattern are observed. In India, Indonesia and Vietnam, the distribution of public subsidy is pro-poor at the level of primary health centre (PHC) and pro-rich at hospitals while in China, Pakistan, Nepal, and Bangladesh it is pro-rich at all levels [20, 26, 27]. In Thailand, Malaysia and Sri Lanka, pro-poor pattern is observed at all level of care [26, 28]. Public subsidy benefits rich more due to its higher utilization by them and due to impediments faced by the poor in availing the services [23, 26, 29].

Studies have used various approaches to understand the impact of public health investment (benefit-incidence analysis, individual preference, concentration curve, and concentration index). Among these, benefit incidence analyses (BIA) is being increasingly used in health economics literature [23, 26–28, 30–33]. Benefit incidence analysis is a tool to access whether the subsidies are helping the poorer section, or the better-off section of the society. It also involves the estimating of the monetary value of the services and their distribution among the population [24]. The analysis helps to capture the effectiveness of the governments in distribution of limited resources to meet the needs of the poor [30].

Over a decade ago, the state of maternal and child health was poor in the country. In 2002–03, the maternal mortality ratio was 286 per 100,000 live births, and the under-five mortality was 74 per 1000 live births [34, 35]. Over half of the mothers did not delivered at a health centre. The prevalence of institutional delivery among women from the poorest wealth quintile was 12.8% compared to 83.6% among those from the richest quintile in 2005 [34]. Inequality was large in the health care utilization [4, 36–38] and the public health subsidies were pro-rich in nature [20]. As a policy response, the Government of India in 2005 revamped the health programme and launched the National Health Mission (NHM), the largest ever health program worldwide. The main objective of the NHM was to improve maternal and child health care in the poorer regions of the country and among the poor and vulnerable sections of the population. The NHM had an estimated annual budget of over ₹26,691 scores in 2017–18, accounting, for more than half of the health budget of the union government [39]. The large-scale public health investments have reduced maternal and child mortality in the country. Deliveries in public health centres has increased from 18% in 2005–06 to 52.1% by 2015–16 [34, 40]. Studies suggest that inequality in health care services has widened across state, rural, and urban areas and wealth quintile [41, 42]. Besides, India, with an UHI service coverage value of 55 is far below the global average of 66 [13]. The slow progress in UHI is associated with high OOP and catastrophic health spending (CHS) [43–46]. About 71% of health spending was met by households in 2004 and 69.1% in 2014 was met by household themselves [47, 48]. OOP is larger in poorer states and among poorer people of poorer states [49]. The catastrophic health spending has shown an increasing pattern, increased from 11.1% in 1995–96 to 24.9% by 2014 [8]. About 3.5% population were impoverished due to medical spending, and about 50.6 million were poor due to medical spending [50].

A number of studies in India have used the BIA approach to examine the benefits of public subsidy on inpatient care, out-patient care, and delivery care. The distribution of public subsidies in Karnataka was six times higher for the richest 20% of the population compared to the poorest 20% [51]. In Northeast India, the benefits of inpatient care were pro-poor in urban and pro rich in rural areas [52]. A recent study found a pro-rich distribution of public subsidy for inpatient care of non-communicable diseases (NCDs) among the elderly [53]. In West Bengal, the benefit of public subsidy was highest for the lower-middle income group in rural areas and for the upper-middle income group in urban areas [23]. During 2004–14, changing pattern of public subsidy for inpatient care was found in Tamil-Nadu, Rajasthan and West Bengal [32]. A recent study suggests that inpatient and delivery services at public health facilities in India are pro-poor [30].

In developing countries, public investment in health has remained low over time and the effectiveness of public spending on healthcare services continue to be an elusive empirical issue. Increasing public health expenditure on health care services does not automatically benefit all groups of the population if the distribution of resources is not equitable [54]. While the average utilization of services may increase, it may not necessarily benefit the poor and the marginalized [55]. Therefore, it is important to empirically assess whether public spending in India truly benefits the poorer section of the population. The national average of the utilisation of delivery care services in public health centres conceals large variations across states and economic groups. Though there has been an increase in the utilization of maternal services in public health centres, little is known as to who is benefiting and it is unclear whether the benefits are largely pro-poor or pro-rich. With this background, we used the BIA and concentration index to examines the equity in the distribution of public subsidy among the mothers using public health centres for institutional delivery.

## Data and methods

Unit data from the most recent round of the National Family Health Survey (NFHS-4) conducted during 2015–16 was used for the analysis. NFHS 4 is the fourth in the series of Demographic Health Survey (DHS) in India that aimed to provide reliable estimates of the utilization of maternal and child health services, contraception, nutrition etc. along with the socio-economic and demographic condition of the households. The NFHS 4 survey used three schedules namely, the household, the women, and the men schedules to collect demographic, health, social and economic information of the household. The household schedule collects information on age, education of members, household amenities, and assets in the household. The women schedule was canvassed to women aged 15–45 years to collect information on such things as fertility, contraception, birth history, ante-natal, natal and post-natal care from sampled households. While information on maternal care services was collected for all the births during the 3 years preceding the survey, information on OOP expenditure on delivery was collected for the last birth in a reference of a five-year periods.

NFHS 4 used multistage stratified sampling using the Census of India, 2011 sampling frame for the selection of the Primary Sampling Units (PSUs). Villages in rural areas and Census Enumeration Blocks (CEBs) in urban areas were used as PSUs. The PSUs were arranged according to female literacy rate and proportion of SC/ST population and were selected using Probability Proportional to Size (PPS) sampling. A complete house listing operation was carried out in each PSU prior to the survey and an average of 22 households were chosen from each selected PSU. The survey successfully interviewed 601,509 households and 699,686 ever married women in the age group 15–49, and 112,122 men in the age group of 15–54 across all states and union territories of India. The NFHS-4 for the first time, included a set of policy-relevant questions on OOP payment on delivery care (defined as the expenditure net of reimbursement) for the last birth delivered at a health centre and reimbursement under the Janani Suraksha Yojana (JSY). Findings of the survey, along with the sampling design, methodology, and results are available in the national report [40].

Unit data from the kids file, which provides details of births to mothers during the 5 years preceding the survey, was also used. A total of 259,627 births were reported of which 190,898 were last births, and 148,645 were conducted in the health centres (institutional delivery). The unit data was cleaned for factual errors on OOP payment before the analysis. The details of and procedures used for data cleaning are available elsewhere [44].

### Statistical analysis

Descriptive statistics, Benefit Incidence Analysis (BIA), and Concentration Index (CI), and Concentration Curve (CC) were used in the analysis.

### Variables

A set of variables including institutional delivery, type of health centre (private/public), level of care at the public health centre (sub-centre [SC], primary health centre [PHC], urban family welfare centre [UFWC], urban primary health centre [UPHC]/government, municipal, rural hospital), OOP payment, place of residence (rural/urban), type of states (low performing / high performing), educational attainment and wealth quintile are used in the analyses. Institutional delivery is defined as the birth of a child at a health centre, classified as either public (government-funded) or private. Care received from Sub-centre, PHC, UHC, UFWC, and UPHC was classified as primary care, while that from government/municipal and rural hospitals was classified as secondary care to allow for a sufficient sample size by each characteristic. The OOP payment, defined as expenditure on delivery care in a health centre without reimbursement was used as the dependent variable. In NFHS 4, the following question on OOP was asked to the mother to estimate there OOP on their last birth “*How much in total did it cost you out of your pocket for this delivery?”*. The OOP was recorded for a five-year period preceding the survey. We have adjusted the OOP to a constant price using a state wise monthly consumer price specific to rural/urban areas. The estimates were provided at 2016 prices. This procedure was used in a recent paper and has been adopted in to derive comparable OOP [44].

The analyses was carried out by characteristics such as rural and urban areas, education (mother’s) of less than five and more than 5 years and low and high performing states (based on the rate of institutional delivery). The economic gradient was measured using wealth index, a composite index based on household assets, durable goods, household amenities etc. In the absence of income or consumption expenditure in the DHS survey, the wealth index is used to measure economic differential in health and health care utilization [40]. In NFHS 4, a set of 43 variables used to derive the wealth index using the principal component analyses (PCA). The wealth index is further classified into five quintile and termed as poorest, poorer, middle, richer and richest. The last birth to a mother, during the 5 years preceding the survey was the unit of analyses.

### Benefit incidence analysis

To determine the distribution of benefits received by various socio-economic groups using public health services for delivery care, Benefit Incidence Analysis has been used. One of the difficulties with benefit incidence analyses is obtaining the cost of services. In the absence of the cost of services, the modal value of OOP payment for delivery has been used in the literature [53]. For our study we used the median value rather than the mean and mode of OOP as a proxy for the cost of services. Like any expenditure data, we found the distribution of OOP to be skewed which made mean unsuitable. Besides, a significant proportion of the mothers had not paid for the services at public/accredited private health centres as they had likely received free services under the Janani Suraksha Yojana Scheme (a demand-side financing scheme for poor mothers in India), making the modal value zero for delivery cost.

The following steps have been used in estimating the benefit-incidence of institutional delivery.

i. Computing wealth quintile (population ranked by wealth) as a measure of socio-economic status.
ii. Estimating the utilization rate for delivery care in public health centres for each quintile.
iii. Estimating net subsidy at public health centres for each quintile (obtained by deducting the median OOP payment on delivery care in public health centres from median OOP payment in private health centres)
iv. Estimating individual subsidy for each quintile by multiplying the net subsidy with the utilization rate.
v. Calculating Benefit Incidence for each quintile by taking percentage share of the individual subsidy.

We estimated the benefit incidence of a particular group j utilizing service i (institutional delivery) in public health centres. The OOP payment in private health centres was taken to be synonymous to the cost of services. Most health insurances in India do not provide any coverage/reimbursement for the maternal care; and so OOP was taken to be equivalent to household expenditure. In case, no charge was levied, the OOP payment was considered zero.

Mathematically, the benefit incidence is defined as follows:

$$ {\mu}_j=\sum {\alpha}_{ij}\frac{\beta_i}{\alpha_i}=\sum {\gamma}_{ij}{\beta}_i $$

Where,

$$ {\upmu}_{\mathrm{j}}=\mathrm{benefit}\ \mathrm{of}\ \mathrm{public}\ \mathrm{subsidy}\ \mathrm{enjoyed}\ \mathrm{by}\ \mathrm{group}\ \mathrm{i} $$

$$ {\upalpha}_{\mathrm{ij}}=\mathrm{utilization}\ \mathrm{of}\ \mathrm{delivery}\ \mathrm{care}\ \left(\mathrm{i}\right)\ \mathrm{by}\ \mathrm{group}\ \mathrm{j} $$

$$ {\upalpha}_{\mathrm{i}}=\mathrm{utilization}\ \mathrm{of}\ \mathrm{delivery}\ \mathrm{care}\ \left(\mathrm{i}\right)\ \mathrm{by}\ \mathrm{all}\ \mathrm{groups} $$

$$ {\upbeta}_{\mathrm{i}}=\mathrm{government}\ \mathrm{net}\ \mathrm{expenditure}\ \mathrm{on}\ \mathrm{delivery}\ \mathrm{care}\ \left(\mathrm{i}\right) $$

$$ {\upgamma}_{\mathrm{ij}}=\mathrm{group}\ \mathrm{j}\ \mathrm{share}\ \mathrm{of}\ \mathrm{utilization}\ \mathrm{of}\ \mathrm{delivery}\ \mathrm{care}\ \left(\mathrm{i}\right) $$

### OOP payment and cost of service on institutional delivery

We computed the OOP payment by wealth quintile for mothers delivering at public health centres. NFHS-4 did not include any information on the actual cost of delivery care at the public health centre. Hence in line with previous literature, we have used the OOP payment for delivery care in private health centres as the proxy for the actual cost of delivery care in public health centres [23, 32].

### Concentration index (CI) and concentration curve (CC)

To examine the economic inequality in the utilization of delivery care services in public/private health centre, we used CC and CI. CC and CI are commonly used by researchers to measure health inequality [56, 57]. CC plots the cumulative proportion of the population (ranked by wealth) against the cumulative proportions of the population utilizing delivery care services in public/private health centres. If CC overlaps with the line of equality, then the extent of utilization of services from public/private health centres is evenly distributed across the wealth group. However, if CC lies above the line of equality, it implies a pro-poor concentration of utilization of delivery care services while if CC lies below the line of equality, it implies a pro-rich concentration in the utilization of delivery care services. CI is defined as twice the area between the CC and the line of equality. The value of CI ranges from − 1 to + 1, with a value of zero suggesting an equal distribution of utilization of services across the wealth group. A negative value signifies a pro-poor distribution of utilization of delivery care services while a positive value signifies a pro-rich distribution [58].

## Result

**Fig. 1:**
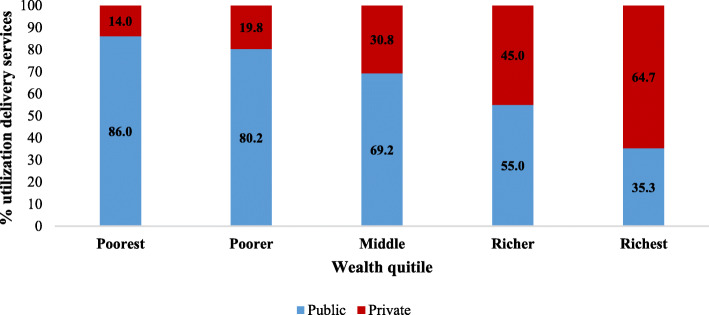
Percentage distribution of institutional delivery by wealth quintile and type of health centre in India, 2015–16

Figure 1 presents the distribution of institutional delivery by wealth quintile and type of health centres in India. The utilization of institutional delivery in public health centres declines with an increase in the economic well-being of the households. On the other hand, the economic gradient in the utilization of institutional delivery in private health centres was strong and positive. For example, among all institutional deliveries in the poorest wealth quintile, 86% were in public health centre compared to 14% in the private ones. By contrast, in the richest wealth quintile, about two-third women used private health centre for delivery care. A majority of the women from the poorest, poorer, and middle quintile availed delivery care in public health centres.

Table 1 presents the socio-demographic characteristics of the study population. About 33% (95% CI: 32.6–33.4) of the respondents resided in urban areas while 67% resides in the rural areas (95% CI: 66.6–67.4). About one-quarter of the respondents had an educational level of less than 5 years (26.9%; 95% CI: 26.6–27.2) while three-fourth of them (73.1, 95% CI: 72.7–73.4) had more than 5 years of education. About 48.8% (95% CI: 48.4–49.2) of the respondents resides in low performing states while 51.2% (95% CI: 50.8–51.6) resided in the high performing ones. With respect to social group, 29.8% (95% CI: 29.5–30.2) of the respondents belonged to schedule caste or schedule tribe, 44.1% (95% CI: 43.7–44.5) belongs to other backward class, and 26.1% belonged to other social groups (95% CI: 25.7–26.4). About 64.7% (95% CI: 64.3–65.1) of the mothers went to public health centres for institutional delivery while, 35.2% of the respondents used private health centres (35.3%; 95% CI: 34.9–35.7). Among respondents utilizing public health centres, 52.8% (95% CI: 52.5–53.2) utilized government/municipal hospitals, rural hospitals while 11.9% (95% CI, 11.6–12.1) utilized Sub-centres, PHC, UHC, others facilities. About 42.5% (95% CI, 42.1–42.9) respondents made less than 4 ANC visit while 57.5% (95% CI, 57.1–57.9) respondents made 4 or more ANC visits.

**Table 1:** Sample profile of the study population based on NFHS-4, 2015–16, India

Variables	Percentage (%)	95% Confidence Interval
**Place of residence**		
Urban	33.0	32.6–33.4
Rural	67.0	66.6–67.4
**Educational Level**		
Less than 5 years	26.9	26.6–27.2
5 years and more	73.1	72.7–73.4
**State type**		
Low performing states	48.8	48.4–49.2
High performing states	51.2	50.8–51.6
**Social Group**		
Schedule caste / Schedule tribe	29.8	29.5–30.2
Other backward class	44.1	43.7–44.5
Others	26.1	25.7–26.4
**Household size**		
Up to 5	47.5	47.1–47.9
6 and more	52.5	52.1–52.9
**Place of Delivery**		
Public facility	64.7	64.3–65.1
Private facility	35.3	34.9–35.7
**Level of care at public health centres**		
Government/Municipal, Rural Hospital	52.8	52.5–53.2
Sub-centre, PHC, UHC, others^a^	11.9	11.6–12.1
**Number of ANC visits**		
Less than 4	42.5	42.1–42.9
4 and more	57.5	57.1–57.9

^a^Others include additional Primary Healthcare Centre (PHC), Urban Health Post (UHP), Urban Family Welfare Centre (UFWC), Public sector health facility

Table 2 presents the percent distribution of women who availed delivery services with and without payment at private and public health centre by wealth quintile in India. About 17% of the respondents did not pay for delivery care in India, and it varies from 15.6% in the poorest wealth quintile to 17.7% in the middle wealth quintile. Among those who availed services in public health centres, the proportion of women who did not pay for delivery care increases by wealth quintile. For example, among respondents who went to primary health centres, 20% in the poorest wealth quintile did not pay for services compared to 30% in the richest wealth quintile. Similarly, among those availing services from secondary health centres, about 16% women in the poorest wealth quintile did not pay for delivery care compared to 28% in the richest wealth quintile. In case of any public health facility, about 17% of the women in poorest wealth quintile did not pay for delivery care compared to 28% in the richest wealth quintile. In the case of private health centres, the proportion of those who did not pay for institutional delivery varied from 7.4% in the poorer quintile to 9.2% in the poorest quintile.

**Table 2:** Percent distribution of mothers who paid and did not pay for institutional delivery by wealth quintile and type of health centres in India, 2015–16

Wealth Quintile	Sub-centres, PHC, UHC & Others^**a**^			Government/Municipal, Rural Hospital			Any public health facility			Private health facility			Overall		
	Paid (%)	Didn’t pay (%)	N	Paid (%)	Didn’t pay (%)	N	Paid (%)	Didn’t pay (%)	N	Paid (%)	Didn’t pay (%)	N	Paid (%)	Didn’t pay (%)	N
Poorest	79.8	20.2	5792	84.4	15.6	18,726	83.4	16.6	24,518	90.8	9.2	3223	84.4	15.6	27,741
Poorer	76.0	24.0	5731	82.1	17.9	20,904	80.9	19.1	26,635	92.6	7.4	5167	83.2	16.8	31,802
Middle	75.5	24.5	4231	78.3	21.7	19,822	77.8	22.2	24,053	92.5	7.5	7838	82.3	17.7	31,891
Richer	70.8	29.2	2511	76.8	23.2	16,165	75.9	24.1	18,676	91.5	8.6	11,149	82.9	17.1	29,825
Richest	70.2	29.8	1161	71.8	28.2	10,572	71.6	28.4	11,733	91.0	9.0	15,653	84.2	15.9	27,386
**Total**	**75.9**	**24.1**	**19,426**	**79.5**	**20.5**	**86,189**	**78.9**	**21.1**	**105,615**	**91.6**	**8.5**	**43,030**	**83.3**	**16.7**	**148,645**

^a^Others include additional Primary Healthcare Centre (PHC), Urban Health Post (UHP), Urban Family Welfare Centre (UFWC), Public sector health facility

Table 3 present the benefit incidence of the public subsidy on delivery care by wealth quintile and level of care in India. The utilization rate in primary health centres varied from 31.9% among the poorest quintile to 6.8% in the richest quintile whereas in secondary health centres, it varied from 23.3% among the poorest quintile to 13.6% in the richest quintile. In case of any public health centre, it varied from 24.8% among the poorest quintile to 12.3% among the richest quintile. By using the overall median OOP payment for service availed in private health centre as the proxy for the cost of services, the public subsidy was found to be pro-poor in each public health facility. During 2015–16, public subsidy in primary health centres was the highest for the poorest quintile (32.29%) followed by the poorer quintile (27.23%) while it was lowest for the richest quintile (6.73%). With regard to secondary health centre, the benefit of public subsidy was maximum for the poorest quintile (23.63%) followed by the poorer quintile (22.55%) while it was the lowest for the richest quintile (13.79%). Considering the quintile specific median cost of service in private health centre, the pattern of the benefit of public subsidy remained similar for primary health centres while different pattern was observed in case of secondary health centre. For instance, in case of any public health centre, the benefit of public subsidy was highest for the middle quintile (21.93%) followed by the richer quintile (21.84%) while it was the lowest for the poorest quintile (17.42%) (Additional file 1).

**Table 3:** Utilization rate, out-of-pocket payment (OOP in US$), and benefit incidence on institutional delivery by wealth quintile and level of care in India, 2015–16

Type of public health centre	Quintile	Number people utilizing public health service (1)	Utilization Rate (2)	Median OOP in public health service in US$ (3)	Median cost of service in private health centre in US$ (4)	Net subsidy at public health centre in US$ (5 = 4–3)	Individual Subsidy Benefit (6 = 5*2)	Benefit Incidence (7)	N
**Primary: Sub-centre, PHC, UHC, & others**^**a**^	**Poorest**	6189	0.319	12	161	150	48	32.29	26,241
	**Poorer**	5323	0.274	15	161	147	40	27.23	24,845
	**Middle**	3986	0.205	15	161	147	30	20.39	22,533
	**Richer**	2612	0.134	15	161	147	20	13.36	18,983
	**Richest**	1316	0.068	15	161	147	10	6.73	13,013
		**19,426**					**148**		**105,615**
**Secondary: Government/ Municipal, Rural Hospital**	**Poorest**	20,052	0.233	15	161	147	34	23.63	26,241
	**Poorer**	19,522	0.227	18	161	144	33	22.55	24,845
	**Middle**	18,547	0.215	18	161	143	31	21.31	22,533
	**Richer**	16,371	0.190	19	161	142	27	18.72	18,983
	**Richest**	11,697	0.136	15	161	147	20	13.79	13,013
		**86,189**					**144**		**105,615**
**Any public health centre**	**Poorest**	26,241	0.248	15	161	147	36	25.10	29,729
	**Poorer**	24,845	0.235	16	161	145	34	23.53	29,729
	**Middle**	22,533	0.213	18	161	144	31	21.12	29,729
	**Richer**	18,983	0.180	18	161	144	26	17.80	29,729
	**Richest**	13,013	0.123	15	161	147	18	12.45	29,729
		**105,615**					**145**		**148,645**

^a^Others include additional Primary Healthcare Centre (PHC), Urban Health Post (UHP), Urban Family Welfare Centre (UFWC), Public sector health facility; 1 US $ = INR 68.22

Table 4 presents the results of the benefit incidence of institutional delivery in India by place of residence, low/high performing states, educational attainment and social group in PHCs, sub-centre, UHCs, and others public health care faculties. The distribution of public subsidy for each of the selected variables was pro-poor in nature. In urban area, the highest share of the benefit was received by women belonging to the poorest quintile (34.39%), followed by those from the poorer quintile (24.93%) while it was the lowest among women from the richest quintile (9.59%). In the case of rural areas, the share of benefit received was highest for women belonging to poorest quintile (27.34%) followed by women from the poorer quintile (24.73%) while it was the lowest among women from the richest quintile (9.76%). The utilization rate in public health facilities of low performing states (LPS) varied from 28.4% among women from the poorest quintile to 8.1% among women from the richest wealth quintile. On the other hand, it varied from 34.4% among those from the poorest quintile to 6.5% among women from the richest quintile in the high performing states (HPS). The share of public subsidy in LPS was highest among the women belonging to the poorest quintile (28.67%) followed by those form the poorer quintile (26.35%), while it was the lowest among the richest quintile (7.99%). In the case of HPS, the share of the benefit was the highest among the poorest quintile (34.44%) followed by poorer (27.02%), while it was the minimum among the richest quintile (6.5%). The utilization rate of public health centres among women with less than 5 years of schooling varied from 25.4% among those from poorest quintile to 11.7% among those from the richest quintile while, it varied from 34.4% among those from the poorest quintile to 6.8% among those from the richest quintile. The share of public subsidy for women with less than 5 years of schooling was highest for those belonging to the poorest quintile (25.79%) followed by the poorer quintile (23.11%), while it was lowest for among women from the richest quintile (11.89%). Among mothers having more than 5 years of education, the share of public subsidy was the highest among the poorest quintile (34.64%), followed by the poorer quintile (26.98%) while it was the lowest for among the richest quintile (6.80%). The utilization pattern and net benefit from public subsidy across social groups by wealth quintile followed a similar pattern; with a higher utilization and greater benefit from seen among mothers belonging to the poorest wealth quintile compared to those from the richest wealth quintile. For example, among mothers belonging to SC/ST, 27.8% of those from the poorest quintile used public services in primary health centres compared to 8.2% of those from the richest quintile. The share of the benefit of public subsidy was also the highest among women from the poorest quintile (28.10%) followed by poorer quintile (25.91%) while it was the lowest among those from the richest quintile (8.05%).

**Table 4:** Utilization rate, out-of-pocket payment (OOP in US$), and benefit incidence by place of residence, educational attainment, states and social group in Sub-centre, PHC, UHC on institutional delivery in India, 2015–16

Sub-centre, PHC, UHC, others^**a**^	Quintile	Number people utilizing public health service (1)	Utilization Rate (2)	Median OOP in public health service in US$ (3)	Median cost of service in private health centre in US$ (4)	Net subsidy at public health centre in US$ (5 = 4–3)	Individual Subsidy Benefit (6 = 5*2)	Benefit Incidence (7)	N
**Urban**	**Poorest**	853	0.343	14	191	177	61	34.39	6789
	**Poorer**	622	0.250	15	191	176	44	24.93	5904
	**Middle**	473	0.190	15	191	176	33	18.99	4818
	**Richer**	303	0.122	16	191	175	21	12.10	3767
	**Richest**	239	0.096	15	191	176	17	9.59	2794
		**2490**					**176**		**24,072**
**Rural**	**Poorest**	4567	0.270	12	147	135	36	27.34	18,905
	**Poorer**	4223	0.249	15	147	132	33	24.73	18,214
	**Middle**	3612	0.213	15	147	132	28	21.16	17,163
	**Richer**	2904	0.171	15	147	132	23	17.01	15,421
	**Richest**	1630	0.096	12	147	132	13	9.76	11,840
		**16,936**					**133**		**81,543**
**LPS**	**Poorest**	3850	0.284	12	142	130	37	28.67	15,983
	**Poorer**	3579	0.264	13	142	129	34	26.35	15,565
	**Middle**	2945	0.217	15	142	127	28	21.44	14,638
	**Richer**	2089	0.154	12	142	130	20	15.56	12,929
	**Richest**	1097	0.081	15	142	127	10	7.99	8767
		**13,560**					**129**		**67,882**
**HPS**	**Poorest**	2020	0.344	15	180	166	57	34.44	9693
	**Poorer**	1585	0.270	15	180	166	45	27.02	8925
	**Middle**	1174	0.200	15	180	166	33	20.01	7925
	**Richer**	706	0.120	15	180	166	20	12.04	6478
	**Richest**	381	0.065	15	180	166	11	6.50	4712
		**5866**					**166**		**37,733**
**Education less than 5 year**	**Poorest**	1866	0.254	10	117	107	27	25.79	7615
	**Poorer**	1719	0.234	13	117	104	24	23.11	7373
	**Middle**	1619	0.221	12	117	105	23	22.06	7210
	**Richer**	1276	0.174	13	117	104	18	17.15	6794
	**Richest**	860	0.117	10	117	107	13	11.89	5815
		**7340**					**105**		**34,807**
**Education more than 5 year**	**Poorest**	4162	0.344	13	176	163	56	34.64	18,360
	**Poorer**	3271	0.271	15	176	161	44	26.98	16,854
	**Middle**	2340	0.194	15	176	161	31	19.30	14,894
	**Richer**	1489	0.123	15	176	161	20	12.28	12,117
	**Richest**	824	0.068	15	176	161	11	6.80	8583
		**12,086**					**162**		**70,808**
**Schedule caste / Schedule tribe**	**Poorest**	2324	0.278	10	147	136	38	28.10	9553
	**Poorer**	2143	0.256	10	147	136	35	25.91	9286
	**Middle**	1856	0.222	12	147	135	30	22.20	8904
	**Richer**	1345	0.161	15	147	132	21	15.74	8237
	**Richest**	688	0.082	15	147	132	11	8.05	6633
		**8356**					**135**		**42,613**
**Other backward class**	**Poorest**	2338	0.313	13	157	144	45	31.60	10,220
	**Poorer**	1920	0.257	15	157	142	37	25.58	9508
	**Middle**	1553	0.208	15	157	142	30	20.69	8462
	**Richer**	1093	0.146	15	157	142	21	14.56	7117
	**Richest**	562	0.075	13	1567	144	11	7.56	4859
		**7466**					**143**		**40,166**
**Other**	**Poorest**	1459	0.405	16	176	160	65	40.54	6247
	**Poorer**	913	0.253	19	176	157	40	24.90	5615
	**Middle**	626	0.174	15	176	161	28	17.55	4736
	**Richer**	389	0.108	16	176	160	17	10.81	3642
	**Richest**	217	0.060	12	176	164	10	6.20	2596
		**3604**					**160**		**22,836**

^a^Others include additional Primary Healthcare Centre (PHC), Urban Health Post (UHP), Urban Family Welfare Centre (UFWC), Public sector health facility; 1 US $ = INR 68.22

Further, the benefit incidence was computed for women using government/municipal hospitals, rural hospitals (Table 5) and any other public health centres (Table 6) for delivery care. The pattern of the distribution of the share of public subsidy in these facilities was similar to that in using PHCs, sub-centres, UHCs and other health facilities; however, the magnitude of the share of the benefit was lower. For instance, in the urban area, among women from the poorest wealth quintile, the share of the benefit of public subsidy was 27.82% among those who went to government/municipal hospitals, rural hospital while it was 28.46% among those availed services from any public health facility compared to 34.39% among those availed services from Sub-centres, PHCs, UHCs, and others.

**Table 5:** Utilization rate, out-of-pocket payment (OOP in US$), and Benefit Incidence by place of residence, educational attainment, states and social group in Government/Municipal, Rural Hospital on institutional delivery in India, 2015–16

Government/ Municipal, Rural Hospital	Quintile	Number people utilizing public health service (1)	Utilization Rate (2)	Median OOP in public health service in US$ (3)	Median cost of service in private health centre in US$ (4)	Net subsidy at public health centre in US$ (5 = 4–3)	Individual Subsidy Benefit (6 = 5*2)	Benefit Incidence (7)	N
**Urban**	**Poorest**	5936	0.275	16	191	174	48	27.82	6789
	**Poorer**	5282	0.245	19	191	172	42	24.34	5904
	**Middle**	4345	0.201	22	191	169	34	19.68	4818
	**Richer**	3464	0.161	18	191	173	28	16.10	3767
	**Richest**	2555	0.118	15	191	176	21	12.07	2794
		**21,582**					**172**		**24,072**
**Rural**	**Poorest**	14,338	0.222	15	147	132	29	22.50	18,905
	**Poorer**	13,991	0.217	16	147	130	28	21.71	18,214
	**Middle**	13,551	0.210	18	147	129	27	20.79	17,163
	**Richer**	12,517	0.194	19	147	128	25	18.99	15,421
	**Richest**	10,210	0.158	15	147	132	21	16.01	11,840
		**64,607**					**130**		**81,543**
**LPS**	**Poorest**	12,133	0.223	15	142	128	28	22.34	15,983
	**Poorer**	11,986	0.221	15	142	128	28	22.06	15,565
	**Middle**	11,693	0.215	15	142	128	27	21.53	14,638
	**Richer**	10,840	0.200	15	142	128	25	19.96	12,929
	**Richest**	7670	0.141	15	142	128	18	14.12	8767
		**54,322**					**128**		**67,882**
**HPS**	**Poorest**	7673	0.241	25	180	155	37	23.79	9693
	**Poorer**	7340	0.230	23	180	157	36	22.97	8925
	**Middle**	6751	0.212	25	180	155	33	20.93	7925
	**Richer**	5772	0.181	23	180	157	28	18.12	6478
	**Richest**	4331	0.136	16	180	164	22	14.19	4712
		**31,867**					**157**		**37,733**
**Education less than 5 years**	**Poorest**	5749	0.209	15	117	103	21	20.93	7615
	**Poorer**	5654	0.206	15	117	103	21	20.58	7373
	**Middle**	5591	0.204	15	117	103	21	20.36	7210
	**Richer**	5518	0.201	15	117	103	21	20.09	6794
	**Richest**	4955	0.180	15	117	103	19	18.04	5815
		**27,467**					**103**		**34,807**
**Education more than 5 year**	**Poorest**	14,198	0.242	18	176	158	38	24.41	18,360
	**Poorer**	13,583	0.231	21	176	155	36	22.92	16,854
	**Middle**	12,554	0.214	22	176	154	33	20.98	14,894
	**Richer**	10,628	0.181	19	176	157	28	18.10	12,117
	**Richest**	7759	0.132	15	176	161	21	13.59	8583
		**58,722**					**157**		**70,808**
**Schedule caste / Schedule tribe**	**Poorest**	7229	0.211	15	147	132	28	21.34	9553
	**Poorer**	7143	0.209	15	147	132	28	21.09	9286
	**Middle**	7048	0.206	18	147	128	26	20.23	8904
	**Richer**	6892	0.201	18	147	129	26	19.89	8237
	**Richest**	5945	0.174	15	147	131	23	17.45	6633
		**34,257**					**130**		**42,613**
**Other backward class**	**Poorest**	7882	0.241	15	157	142	34	24.25	10,220
	**Poorer**	7588	0.232	15	157	142	33	23.58	9508
	**Middle**	6909	0.211	16	157	141	30	21.04	8462
	**Richer**	6024	0.184	18	157	139	26	18.15	7117
	**Richest**	4297	0.131	15	157	142	19	13.22	4859
		**32,700**					**141.3**		**40,166**
**Others**	**Poorest**	4788	0.249	22	176	154	38	24.96	6247
	**Poorer**	4702	0.244	26	176	150	37	23.92	5615
	**Middle**	4110	0.214	23	176	152	33	21.26	4736
	**Richer**	3253	0.169	23	176	153	26	16.91	3642
	**Richest**	2379	0.124	15	176	161	20	12.96	2596
		**19,232**					**153**		**22,836**

1 US $ = INR 68.22

**Table 6:** Utilization rate, out-of-pocket payment (OOP in US$), and Benefit incidence place of residence, educational attainment, states and social group on institutional delivery by wealth quintile in India, 2015–16

Overall	Quintile	Number people utilizing public health service (1)	Utilization Rate (2)	Median OOP in public health service in US$ (3)	Median cost of service in private health centre in US$ (4)	Net subsidy at public health centre in US$ (5 = 4–3)	Individual Subsidy Benefit (6 = 5*2)	Benefit Incidence (7)	N
**Urban**	**Poorest**	6789	0.282	16	191	175	49	28.46	8461
	**Poorer**	5904	0.245	18	191	173	42	24.46	8460
	**Middle**	4818	0.200	20	191	171	34	19.69	8460
	**Richer**	3767	0.156	18	191	173	27	15.61	8460
	**Richest**	2794	0.116	15	191	176	20	11.77	8460
		**24,072**					**173**		**42,301**
**Rural**	**Poorest**	18,905	0.232	15	147	132	31	23.38	21,269
	**Poorer**	18,214	0.223	15	147	131	29	22.40	21,270
	**Middle**	17,163	0.210	17	147	130	27	20.92	21,268
	**Richer**	15,421	0.189	18	147	129	24	18.65	21,269
	**Richest**	11,840	0.145	15	147	132	19	14.64	21,268
		**81,543**					**131**		**106,344**
**LPS**	**Poorest**	15,983	0.235	15	142	128	30	23.55	17,852
	**Poorer**	15,565	0.229	15	142	128	29	22.93	17,852
	**Middle**	14,638	0.216	15	142	128	27	21.56	17,853
	**Richer**	12,929	0.190	15	142	128	24	19.05	17,851
	**Richest**	8767	0.129	15	142	128	16	12.92	17,851
		**67,882**					**128**		**89,259**
**HPS**	**Poorest**	9693	0.257	22	180	158	41	25.59	11,878
	**Poorer**	8925	0.237	22	180	158	37	23.57	11,877
	**Middle**	7925	0.210	23	180	158	33	20.83	11,877
	**Richer**	6478	0.172	22	180	158	27	17.11	11,877
	**Richest**	4712	0.125	16	180	158	21	12.90	11,877
		**37,733**					**159**		**59,386**
**Education less than 5 years**	**Poorest**	7615	0.219	14	117	103	23	22.0	8410
	**Poorer**	7373	0.212	15	117	103	22	21.15	8411
	**Middle**	7210	0.207	15	117	103	21	20.68	8408
	**Richer**	6794	0.195	15	117	103	20	19.49	8410
	**Richest**	5815	0.167	15	117	103	17	16.68	8409
		**34,807**					**103**		**42,048**
**Education more than 5 years**	**Poorest**	18,360	0.259	16	176	160	41	26.18	21,320
	**Poorer**	16,854	0.238	18	176	158	37	23.70	21,320
	**Middle**	14,894	0.210	21	176	155	33	20.65	21,321
	**Richer**	12,117	0.171	18	176	158	27	17.12	21,317
	**Richest**	8583	0.121	15	176	161	20	12.35	21,319
		**70,808**					**158**		**106,597**
**Schedule caste/ Schedule**	**Poorest**	9553	0.224	13	147	133	30	22.72	10,417
	**Poorer**	9286	0.218	15	147	132	29	21.84	10,417
	**Middle**	8904	0.209	16	147	130	27	20.69	10,417
	**Richer**	8237	0.193	16	147	130	25	19.16	10,417
	**Richest**	6633	0.156	15	147	132	21	15.60	10,417
		**42,613**					**132**		**52,085**
**Other backward class**	**Poorest**	10,220	0.254	15	157	142	36	25.55	12,048
	**Poorer**	9508	0.237	15	157	142	34	23.77	12,048
	**Middle**	8462	0.211	16	157	141	30	20.93	12,048
	**Richer**	7117	0.177	16	157	141	25	17.61	12,048
	**Richest**	4859	0.121	15	157	142	17	12.15	12,048
		**40,166**					**142**		**60,240**
**Others**	**Poorest**	6247	0.274	22	176	154	42	27.29	7264
	**Poorer**	5615	0.246	23	176	152	37	24.29	7264
	**Middle**	4736	0.207	22	176	154	32	20.69	7264
	**Richer**	3642	0.159	22	176	154	25	15.91	7264
	**Richest**	2596	0.114	15	176	161	18	11.83	7264
		**22,836**					**154**		**36,320**

1 US $ = INR 68.22

**Fig. 2:**
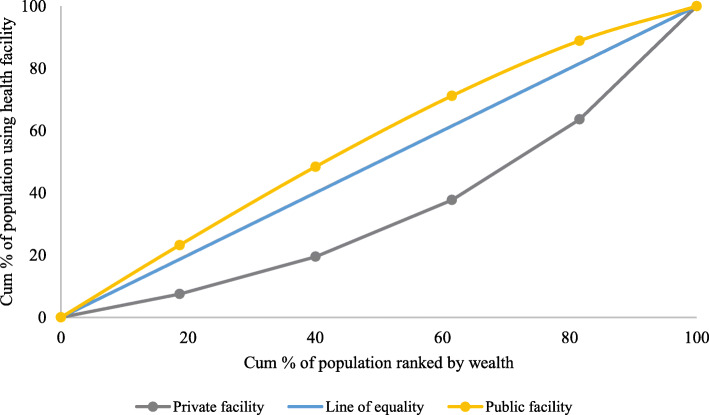
Concentration curve for mothers using delivery services at public and private health facility in India, 2015–16

Figure 2 present the concentration curve (CC) for women who had institutional delivery at public and private health centres. The CC for women who went to public health centre is above the line of equality, indicating a pro-poor concentration of the of public health centre for delivery care services whereas CC is below the line of equality for women who went to private health centre suggesting a pro-rich concentration of the use of private health centres for delivery care services.

Table 7 presents the concentration index for institutional delivery by place of residence, low/high performing states, educational attainment, household size, number of ANC visits and adverse birth outcome in India by use of services in public and private health centres. For women who went to public health centres, the CI value was negative for each of the selected variable, suggesting pro-a pro-poor utilization of services while was pro-rich for those who went to private health centres. The CI values was higher for women resided in urban areas and used a public health centre (CI: − 0.209) for delivery care compared to those who delivered in a private health centre (CI: − 0.112). Similarly, the CI was higher for mother who used private health centres for delivery services and were from rural area (CI: 0.281) compared to those form urban areas (CI: 0.217). The CI value of was higher for women resided in an HPS (− 0.177) compared to those to those resided in an LPS (− 0.113). On the contrary, in the case of private health centre the CI value was higher for women who resided in an LPS (0.318) compared to those who resided in an HPS (0.226). In the terms of education the CI value was higher in the case of women who used the public health care services had more than 5 years of education (− 0.177) compared to those who had having less than 5 years of education (− 0.063). Similarly, In the case of private health centre too, the CI value was higher among mothers having an education of more than 5 years (0.258) compared to those having less than 5 years of education (0.240). The CI value was higher for women who made 4 or more ANC visit (− 0.184) used public health services compared to those who made less than 4 ANC visit (− 0.107). Conversely, in the case of private health care centres the CI value was higher for women who made less than 4 or more ANC visits (0.298) utilizing private health centres compared to those having less than 4 ANC visits (0.257). The CI value was lower for women who had an adverse birth outcome (− 0.150) and used a public health facility compared to those who did not have adverse birth outcome (− 0.166). Similarly, in the case of private health facilities the CI value was lower for women who had an adverse birth outcome (0.280) compared to those who did not (0.302).

**Table 7:** Concentration index for institutional delivery by selected covariates in India, 2015–16

	Place of Delivery			
	Public	95% Confidence Interval	Private	95% Confidence Interval
**Place of Residence**				
Rural	−0.112	(−0.115, −0.109)	0.281	(0.273, 0.290)
Urban	−0.209	(−0.218, −0.200	0.217	(0.207, 0.226)
**State type**				
Low Performing States	−0.113	(−0.116, −0.110)	0.318	(0.309, 0.328)
High Performing state	−0.177	(−0.184, −0.170)	0.226	(0.217, 0.235)
**Education**				
Less than 5 years	−0.063	(−0.067, −0.058)	0.24	(0.222, 0.258)
5 years and more	−0.177	(−0.182, −0.172)	0.258	(0.251, 0.265)
**Household Size**				
Up to 5	− 0.167	(−.0172, − 0.162)	0.307	(−.0299, 0.315)
6 or more	−0.152	(−.0157, − 0.147)	0.277	(−.0267, 0.287)
**Number of ANC visits**				
Less than 4	−0.107	(−0.112, − 0.102)	0.298	(0.285, 0.311)
4 and more	−0.184	(−0.189, − 0.179)	0.257	(0.249, 0.265)
**Adverse Birth Outcome**				
No	−0.166	(−0.170, − 0.162)	0.302	(0.294, 0.310)
Yes	−0.150	(−0.156, − 0.144)	0.280	(0.269, 0.291)
**Overall**	−0.161	(−0.165, − 0.158)	0.296	(0.289, 0.303)

**Fig. 3:**
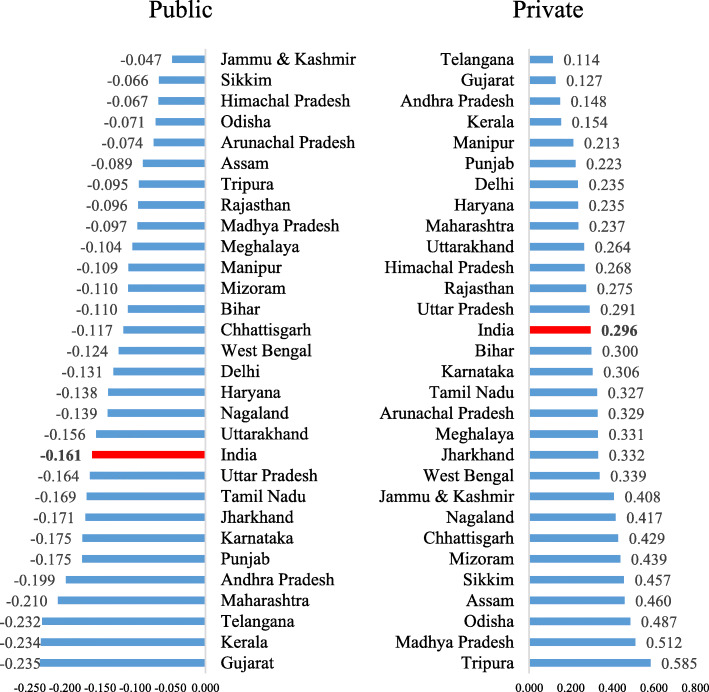
Concentration Index of institutional delivery by public and private facility in selected states of India, 2015–16

Figure 3 represents the concentration index for delivery care across the states of India by public and private health facilities. The CI value for mothers who used public health centres was − 0.161 and negative for all the states. In contrast, the CI value for mothers who used private health centres was 0.296 and positive for all the states. A large variation in concentration index was observed across the states for both public and private health facilities. In the case of public health facilities, the CI value was the highest in Gujarat (CI: − 0.235) followed Kerala (CI: − 0.234) and Telangana (CI: − 0.232) and the lowest in Jammu & Kashmir (CI: − 0.047) followed by Sikkim (CI: − 0.066) and Himachal Pradesh (− 0.067). Across private health centres, the CI value was the highest in Tripura (CI: 0.585) followed by Madhya Pradesh (0.512) and Odisha (0.487) and the lowest in Telangana (CI: 0.114), followed by Gujarat (CI: 0.127) and Andhra Pradesh (CI: 0.148).

## Discussion

Resource constraints are one of the major challenges faced by the public healthcare system in developing countries. Resources used for public health services have an opportunity cost, and in this context equity in health care is assumed to be significant. The NHM in India, the largest ever public health programmes worldwide has been operational for over 15 years. About half of the national health resources are invested in NHM, with the aim of achieving multiple objectives including increasing service coverage, reducing inequality in health care and health outcomes and reducing OOP payment and CHS specifically among the poor and the disadvantaged. The priorities of these schemes are usually to benefit the economically weaker section of the population, and studies attributed to increase in maternal care utilization and improvement in health outcome to the NHM [59–62]. There are limited studies on the distributional aspect of public subsidy on health care utilisation in India. This study using the latest and largest-ever nationwide population-based survey data examine the distribution of public subsidy among mothers using primary and secondary public health centres considering institutional delivery as the case. The salient findings of the paper are as follows:

First, the utilization of delivery care in the public health centres is pro-poor. Mothers belonging to the poorest and poorer wealth quintile use more of the public health centre for delivery care while mothers from the richer and the richest wealth quintile use more of the private health centre for delivery care services. Second, the distribution of public subsidy for institutional delivery in both primary and secondary public health centre are pro-poor and the gradient is stronger in primary health centre compared to secondary health centres. About 32% of net subsidy were availed among women of the poorest wealth quintile and using primary health centres compared to 24% for women belonging to the poorest wealth quintile and who went to secondary health centres. Our findings regarding the subsidy being pro poor at the primary health centre is robust even through the use of alternative cost measures (quintile specific cost in private health centres). Third, the share of public subsidy is pro-poor in nature for each of the selected co-variates such as rural/urban, social class, and LPS/HPS across primary and secondary levels of care. However, within the same wealth quintile, we found a higher gradient in the use of services and the net benefit of subsidy among mother with higher educational attainment than those with lower educational attainment. Fourth, the concentration curve for mother using public health centres for delivery care was above the line of equality suggesting a pro-poor concentration of use of public health service on the other hand the curve was below the line of equality suggesting a pro-rich concentration of use of private health services. The CI value of − 0.161 for public health centres and 0.296 for private health centres further confirms the concentration of use public health centres among the poor and private health centres among the rich. The state variation in the concentration index ware large for both public and private health services.

We provide some plausible explanations for our findings. The use of delivery care in public health centres is higher among the poorest and the poorer section of the population as public health centres are provided at free or very nominal cost and poor people has limited ability to pay for services. These findings may be due to implementation of JSY and other schemes under NHM that led to increase in utilization of maternal services [30, 60, 61, 63]. The trend of pro-poor utilization of public health facilities in India is consistent with literatures. For example using the NSS 71st round data [30] showed with the help of concentration index (CI) that public service utilization at the national level is pro-poor for both inpatients and delivery care. The institutional delivery in private health centres is expensive and the services are mostly used by the richer and the richest wealth quintile. The OOP payment during delivery may be on the account of the complications in delivery care, caesarean delivery, cost of medicine, transportation costs and costs related to the and accompanying person, and has a strong and positive economic gradient. Mothers from the higher economic strata have a higher ability to pay for services and so they seek for better quality of care [64]. Our key findings regarding the net subsidy on institutional delivery being pro-poor in nature at primary and secondary health centre may be attributed to the provisioning of cash incentives and facilities under JSY and state-specific schemes. About two decades ago the hospitalization and outpatient services were pro-rich over the time, the tends have reversed [20, 30]. Though our result about the pro-poor nature of the subsidy holds true for primary health centre even after using quintile specific costs, it does not hold true for secondary health centres. Although, the marginalized women should receive reimbursements and incentives from NHM and other maternal programmes, studies suggest that, these incentives are either insufficient or there are some other factors accounting for the inequality, such as, low education attainment, and low quality of the public health facilities in poorer areas [26, 65]. Regional variation in subsidy utilization can be another possible reason behind the unequal distribution of public subsidies. For instance, poor mothers from the LPS avail the benefit of subsidy which can be explained by the introduction of various maternal and child health programmes under NHM. Although inequality still exists, the level of inequality has reduced significantly across all groups in LPS [61, 65, 66]. Besides increasing facility based delivery, JSY has significantly increased contraceptive use, breastfeeding practice and post-natal check-up, all of which are closely associated with accessing public health facilities [60, 63]. The *Ayushman Bharat* scheme, that was launched by the Government of India in 2018 will provide further financial protection for the use of health services to 500 million people; accounting for 40% of the population of India in a phased manner. The scheme offers cashless payment for hospitalization to empanelled public and private hospitals covering an expenditure of US$ 7329 (Rs.500,000) per family per year. It is the largest ever public sponsored insurance scheme worldwide and is operational in many states of India. As of October 25th, 2020, more than 12 scores people have already benefited from the scheme. Other such initiative include the *Pradhan Mantri Matru Vandana Yojana (PMMVY)* which offers a cash incentive of US$ 73(Rs.5000) to pregnant and lactating mothers of age 19 years and above for their first live birth.

Our findings have the following implications. First, we suggest improving the physical infrastructure and service coverage in the public health centres, particularly the primary health centres. Our findings demonstrate a higher use of services and net subsidy at these centres by the poorest and the poorer sections of the population. But the primary health centres are equipped with limited services and infrastructure. A PHC constitutes an inpatient ward area with 4 to 6 beds, a labour room, and a minor operation theatre for a population of 20,000 to 30,000 based on the type of area [67] and is not equipped to conduct caesarean or complicated deliveries. The treatment availability of preventive services in PHCs is very minimal. It has been found that the utilization rate of public facilities from secondary level among the richer 40% of the population is more than that of primary level. This indicates that the richer section demand more public facilities at the secondary level. One potential reason behind this can be the better quality of care at the secondary level which attracts them to utilize the public health facilities. Besides, there may be certain impediment for the poor people to access secondary services. From the policy perspective, there is need for more equal and more efficient allocation of public spending at the primary level is required. At the secondary level, improving the quality of services and extending service coverage to non-communicable diseases is recommended. Addressing the impediments faced by the poor in availing quality services, particularly, caesarean and complicated delivery in public health centres may be considered. Implementing, these steps may help to reduce the high OOP payment and CHS among the poor and achieve equity in access to delivery care in India. Overall, there is need to improve the quality of care in public health centres to overcome geographical barriers in remote areas.

We outline the following limitations of the study. First, since we used self-reported data from the NFHS to estimate utilization pattern, OOP payments, and benefit incidence, there may have been be some recall bias. Besides, the indirect cost associated with institutional delivery was not covered in the survey. Second, we used the median cost of services in private health centres as a proxy for the cost of services in public health centres. An appropriate study on costing may provide more robust to bring out the actual scenario. Third, our results could not cover the impact of recent initiatives such Ayushmann Bharat, and the Pradhan Mantri Matru Vandana Yojana. As these were launched after the completion of the NFHS 4. Such analyses may be feasible with the release of the fifth round of the NFHS.

## Conclusion

Public health spending should benefit the poor and the marginalized section of the society to achieve equity in health outcomes. At the national level, policies such as, the Rashtriya Swasthya Bima Yojana (RSBY), Ayushman Bharat, and the Pradhan Mantri Matru Vandana Yojana (PMMVY) have been providing protection against financial risks to the economically weaker section of the population. These policies are significant to change the very outline of health care access, utilization of services, and OOP expenditure. It is recommended to continue these programmes with greater monitoring surveillance to make them more pro-poor, so that the disadvantaged section of the population can receive the necessary support. Investing in the public health infrastructure and improving the quality of services in primary and secondary health centre is recommended.

## Supplementary Information

**Additional file 1.** Utilization rate, out-of-pocket payment (OOP in US$), and Benefit incidence on institutional delivery by wealth quintile and level of care in public health centres using quintile specific OOP in private health centres as proxy to cost of services in public health centres of India, 2015–16

## Data Availability

The dataset used and analysed for the current study is available in DHS repository, [https://dhsprogram.com/data/dataset/India_Standard-DHS_2015.cfm?flag=0].

## Abbreviations

NHM: National Health Mission
NFHS: National Family Health Survey
OOP: Out-of-Pocket Payment
UHC: Urban Health Centre
UHP: Urban Health Post
UFWC: Urban Family Welfare Centre
BIA: Benefit Incidence Analysis
CI: Concentration Index
CC: Concentration Curve
CHS: Catastrophic Health Spending
MDG: Millennium Development Goals
SDG: Sustainable Development Goals
UHI: Universal Health index
PHC: Primary Health Centre
NCD: Non-Communicable Diseases
DHS: Demographic Health Survey
CEB: Census Enumeration Blocks
PPS: Probability Proportional to Size
UPHC: Urban Primary Health Centre
PCA: Principal Component Analysis
JSY: Janani Suraksha Yojana
LPS: Low Performing States
HPS: High Performing States
RSBY: Rashtriya Swasthya Bima Yojana
