# Supplementary material for: Understanding equity of institutional delivery in public health centre by level of care in India: an assessment using benefit incidence analysis
Source: Int J Equity Health. 2020 Dec 9;19:217. doi: 10.1186/s12939-020-01331-z (PMC7724812; doi:10.1186/s12939-020-01331-z)
Supplement: Supplementary file 1 — Additional file 1. Utilization rate, out-of-pocket payment (OOP in US$), and Benefit incidence on institutional delivery by wealth quintile and level of care in public health centres using quintile specific OOP in private health centres as proxy to cost of services in public health centres of India, 2015–16 [file 12939_2020_1331_MOESM1_ESM.docx]

**Supplement Table**

**Appendix 1:** Utilization rate, out-of-pocket payment (OOP in US$), and Benefit incidence on institutional delivery by wealth quintile and level of care in public health centres using quintile specific OOP in private health centres as proxy to cost of services in public health centres of India, 2015-16

| **Type of public health centre** | **Quintile** | **Number people utilizing public health service (1)** | **Utilization Rate (2)** | **Median OOP in public health service (3)** | **Median cost of service in private health centre (4)** | **Net subsidy at public health centre (5=4-3)** | **Individual Subsidy Benefit (6=5*2)** | **Benefit Incidence (7)** | **N** |
| --- | --- | --- | --- | --- | --- | --- | --- | --- | --- |
| **Sub-centre, PHC, UHC, others^*^** | Poorest | 6189 | 0.319 | 12 | 103 | 91 | 29 | 24.30 | 26241 |
|  | Poorer | 5323 | 0.274 | 15 | 120 | 106 | 29 | 24.27 | 24845 |
|  | Middle | 3986 | 0.205 | 15 | 147 | 132 | 27 | 22.72 | 22533 |
|  | Richer | 2612 | 0.134 | 15 | 170 | 155 | 21 | 17.53 | 18983 |
|  | Richest | 1316 | 0.068 | 15 | 211 | 196 | 13 | 11.17 | 13013 |
|  |  | **19426** |  |  |  |  | **119** |  | **105615** |
| **Government/Municipal, Rural Hospital** | Poorest | 20052 | 0.233 | 15 | 103 | 88 | 20 | 16.16 | 26241 |
|  | Poorer | 19522 | 0.227 | 18 | 120 | 103 | 23 | 18.36 | 24845 |
|  | Middle | 18547 | 0.215 | 18 | 147 | 128 | 28 | 21.79 | 22533 |
|  | Richer | 16371 | 0.19 | 19 | 170 | 151 | 29 | 22.64 | 18983 |
|  | Richest | 11697 | 0.136 | 15 | 211 | 196 | 27 | 21.05 | 13013 |
|  |  | **86189** |  |  |  |  | **127** |  | **105615** |
| **Any public**  **health centre** | Poorest | 26241 | 0.248 | 15 | 103 | 88 | 22 | 17.42 | 29729 |
|  | Poorer | 24845 | 0.235 | 16 | 120 | 104 | 24 | 19.51 | 29729 |
|  | Middle | 22533 | 0.213 | 18 | 147 | 129 | 28 | 21.93 | 29729 |
|  | Richer | 18983 | 0.18 | 18 | 170 | 152 | 27 | 21.84 | 29729 |
|  | Richest | 13013 | 0.123 | 15 | 211 | 196 | 24 | 19.29 | 29729 |
|  |  | **105615** |  |  |  |  | **125** |  | **148645** |

**Others include additional Primary Healthcare Centre (PHC), Urban Health Post (UHP), Urban Family Welfare Centre (UFWC), Public sector health facility; 1 US $=INR 68.22*
